# Involvement of the Histone-Like Nucleoid Structuring Protein (H-NS) in *Acinetobacter baumannii’s* Natural Transformation

**DOI:** 10.3390/pathogens10091083

**Published:** 2021-08-26

**Authors:** Casin Le, Camila Pimentel, Marisel R. Tuttobene, Tomás Subils, Jenny Escalante, Brent Nishimura, Susana Arriaga, Deja Rodgers, Robert A. Bonomo, Rodrigo Sieira, Marcelo E. Tolmasky, María Soledad Ramírez

**Affiliations:** 1 Center for Applied Biotechnology Studies, Department of Biological Science, College of Natural Sciences and Mathematics, California State University Fullerton, Fullerton, CA 92831-3599, USA; thanhle1998@csu.fullerton.edu (C.L.); camilapimentel99@csu.fullerton.edu (C.P.); jenni1@csu.fullerton.edu (J.E.); bnish-942@csu.fullerton.edu (B.N.); arriagasusie@gmail.com (S.A.); deja.rodgers@gmail.com (D.R.); mtolmasky@fullerton.edu (M.E.T.); 2 Área Biología Molecular, Facultad de Ciencias Bioquímicas y Farmacéuticas, Universidad Nacional de Rosario, Rosario S2002LRK, Argentina; tuttobene@ibr-conicet.gov.ar; 3 Instituto de Biología Molecular y Celular de Rosario (IBR, CONICET-UNR), Rosario S2002LRK, Argentina; 4 Instituto de Procesos Biotecnológicos y Químicos de Rosario (IPROBYQ, CONICET-UNR), Rosario S2002LRK, Argentina; subils@iprobyq-conicet.gob.ar; 5 Research Service and GRECC, Louis Stokes Cleveland Department of Veterans Affairs Medical Center, Cleveland, OH 44106, USA; Robert.Bonomo@va.gov; 6 Departments of Medicine, Pharmacology, Molecular Biology and Microbiology, Biochemistry, Proteomics and Bioinformatics, Case Western Reserve University School of Medicine, Cleveland, OH 44106, USA; 7 CWRU-Cleveland VAMC Center for Antimicrobial Resistance and Epidemiology (Case VA CARES), Cleveland, OH 44106, USA; 8 Fundación Instituto Leloir—IIBBA CONICET, Buenos Aires C1405BWE, Argentina; rsieira@leloir.org.ar

**Keywords:** *Acinetobacter baumannii*, H-NS, natural transformation, naturally competent, DNA acquisition

## Abstract

Most *Acinetobacter baumannii* strains are naturally competent. Although some information is available about factors that enhance or reduce the frequency of the transformation of this bacterium, the regulatory elements and mechanisms are barely understood. In this article, we describe studies on the role of the histone-like nucleoid structuring protein, H-NS, in the regulation of the expression of genes related to natural competency and the ability to uptake foreign DNA. The expression levels of the natural transformation-related genes *pilA*, *pilT*, *pilQ*, *comEA*, *comEC*, *comF*, and *drpA* significantly increased in a Δ*hns* derivative of *A. baumannii* A118. The complementation of the mutant with a recombinant plasmid harboring *hns* restored the expression levels of six of these genes (*pilT* remained expressed at high levels) to those of the wild-type strain. The transformation frequency of the *A. baumannii* A118 Δ*hns* strain was significantly higher than that of the wild-type. Similar, albeit not identical, there were consequences when *hns* was deleted from the hypervirulent *A. baumannii* AB5075 strain. In the AB5075 complemented strain, the reduction in gene expression in a few cases was not so pronounced that it reached wild-type levels, and the expression of *comEA* was enhanced further. In conclusion, the expression of all seven transformation-related genes was enhanced after deleting *hns* in *A. baumannii* A118 and AB5075, and these modifications were accompanied by an increase in the cells’ transformability. The results highlight a role of H-NS in *A. baumannii’s* natural competence.

## 1. Introduction

The histone-like nucleoid structuring protein (H-NS) is a global regulator, widely distributed among different genera of bacteria. H-NS functions to directly repress transcription across the genome. H-NS-like proteins are shown to assist horizontal DNA transmission and have important implications for bacterial evolution [1]. In *Enterobacteriaceae*, H-NS acts as a transcriptional repressor of the type I-E CRISPR-Cas system leading to natural transformation events [2,3]. A correlation between H-NS-mediated regulation and the lack of conservation of the respective potential horizontally acquired gene clusters in different *Acinetobacter* sp. genomes was observed. This evidence indicated that H-NS acts as a xenogenic repressor in *A. baumannii* [4]. *A. baumannii* H-NS disruption is known to regulate genes associated with quorum sensing, type VI secretion system, type I pili, phenylacetic acid degradation, and acetoin metabolism, among other functions [4]. Horizontal gene transfer (HGT) mechanisms play a crucial role in the dissemination of antimicrobial resistance [5,6]. Natural transformation, one of the main HGT mechanisms that promotes the integration of exogenous DNA, has been documented in approximately 80 bacterial species [5,7]. Many of the *Acinetobacter* sp. are naturally competent, making transformation a critical strategy for evolution and acquiring novel genetic material [8,9,10,11,12,13,14,15,16,17,18,19,20]. *A. baumannii’s* genomes are highly variable, showing large segments of DNA of different origins, which often code for virulence factors, adaptability systems, and antibiotic resistance [21,22,23,24].

The transformation frequency of *A. baumannii* increases in the presence of human pleural fluid and human serum albumin [13,20,25]. Furthermore, *A. baumannii* DNA uptake occurs while moving across wet surfaces [12]. Further studies refined our understanding of natural competency, which correlates with the growth phase-dependent synthesis of a type IV pilus [26]. The studies described above conclusively show that motility and natural competence are intimately associated. As a result, it is possible that other factors affecting motility also impact the capability of *A. baumannii* to take up DNA. The recent report that the disruption of the *A. baumannii* ATCC 17978 *hns* gene by an insertion sequence result in hyper-motility [27], as well as the role of H-NS in genome stability [1,2] raised the question that an H-NS function may also be associated with the natural transformation in *A. baumannii*. This analysis describes the significant enhancement in expression levels of genes related to natural competence when *hns* is deleted.

## 2. Results and Discussion

### 2.1. H-NS Role in Natural Transformation in the First Naturally Competent A. baumannii Clinical Isolate

To determine the effect of the H-NS global regulator in natural transformation, we deleted the genes in two experimentally validated *A. baumannii* strains and determined the expression of competence-associated genes. Pilus-related genes and twitching motility are essential for *A. baumannii’s* transformability [8,26]. Therefore, we compared the expression levels of *pilA*, *pilT*, *pilQ*, *comEA*, *comEC*, *comF*, and *drpA* in the wild type, the *Δhns*, and a complemented strain. The latter was constructed by introducing the *hns*-carrying plasmid pMBLe-*hns* into the *Δhns* mutant.

**Figure 1:**
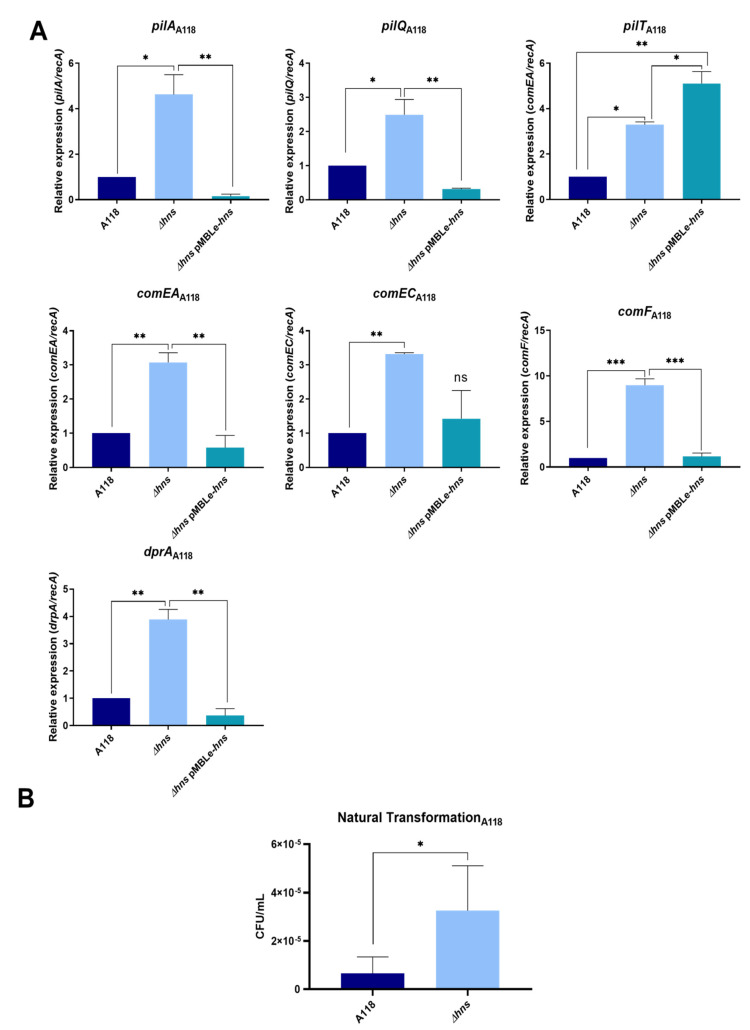
(**A**) The qRT-PCR of *A. baumannii* A118, A118 *Δhns* and A118 *Δhns* pMBLe-*hns* genes associated with competence and type IV pilus: *pilA*, *pilQ*, *pilT*, *comEA*, *comEC*, *comF* and *dprA*. Fold changes were calculated using double Δ*Ct* analysis. At least three independent samples were tested, and three technical replicates were performed from each sample. Statistical significance (* *p* < 0.05) was determined by ANOVA followed by Tukey’s comparison test; one asterisk: * *p* < 0.05; two asterisks: ** *p* < 0.01 and three asterisks: *** *p* < 0.001. (**B**) Natural transformation frequencies for *A. baumannii* A118 and A118 *Δhns* strains in LB broth. At least three independent replicates were performed and *p* < 0.05 was considered significant (*t* test).

Quantitative RT-PCR (qRT-PCR) assays using total RNA demonstrated that the expression levels of all tested genes were significantly increased (Figure 1A). Exceptionally, significant differences were not observed between *Δhns* and *Δhns* pMBLe-*hns* for the *comEC* gene in *A. baumannii* A118. Furthermore, except for *pilT*, the expression levels of these genes in the complemented mutant were reduced to wild-type levels (Figure 1A). Consistent with these results, the assessment of the wild-type and the *Δhns* mutant transformation frequencies showed a five-fold increase in *A. baumannii* A118 *Δhns* (*p* < 0.05) (Figure 1B).

### 2.2. The Expression of Natural Competence Associated Genes Is also under the Control of H-NS in a Hypervirulent and Resistant Model Strains

**Figure 2:**
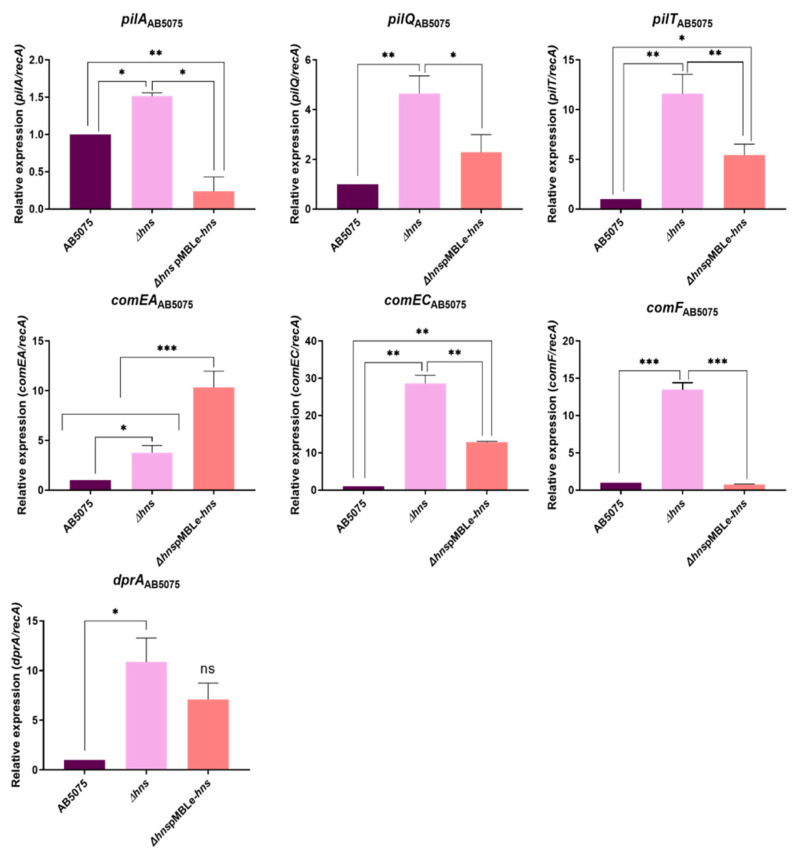
qRT-PCR of *A. baumannii* AB5075, AB5075 *Δhns* and AB5075 *Δhns* pMBLe-*hns* genes associated with competence and type IV pilus: *pilA*, *pilQ*, *pilT*, *comEA*, *comEC*, *comF* and *dprA*. Fold changes were calculated using double ΔCt analysis. At least three independent samples were used, and three technical replicates were performed from each sample. Statistical significance (* *p* < 0.05) was determined by ANOVA followed by Tukey’s comparison test; one asterisks: * *p* < 0.05; two asterisks: ** *p* < 0.01 and three asterisks: *** *p* < 0.001.

The effect of H-NS in natural competence was also studied in the hypervirulent *A. baumannii* AB5075, its *Δhns* derivative, and a complemented strain carrying pMBLe-*hns*. As was the case for *A. baumannii* A118 and A118 *Δhns*, all seven genes, *pilA*, *pilT*, *pilQ*, *comEA*, *comEC*, *comF*, and *drpA*, were expressed at higher levels in the mutant (Figure 2). The complementation via the introduction of pMBLe-*hns* caused a reduction in the expression levels in six genes. The expression of *comEA* was the only exception to this behavior. The levels of expression of the *comEC* gene in the absence of H-NS were higher with respect to the other genes; this may have been due to the essential *comEC* function. In natural transformation, the single-stranded DNA translocated across the inner membrane through the *comEC* channel [26]. Underscoring the importance of *comEC* in DNA acquisition, other inner-membrane DNA translocation channels have not been described to date. It is common that essential functions are equipped to be expressed at levels much higher than needed, but tight mechanisms negatively regulate them. When H-NS is deleted, the high levels of expression of *comEC*, are potentially one of these cases. *comEC* may be a target of interest as we observed a slight reduction in the expression of this gene after complementation in both strains.

The levels of expression of *comEA* gene in AB5075 *Δhns* pMBLe-*hns* could play a role in virulence in this strain since this result was not observed in the A118 complemented strain, and the virulence and susceptibility profile were different for both strains. As was the case with *pilA* in the *A. baumannii* A118 *Δhns* (pMBLe-*hns*), we still do not know the significance and impact of these responses to the production of H-NS from an extrachromosomal element. The natural transformation frequency of *A. baumannii* AB5075 *Δhns* was five-fold higher than that of the AB5075 parent strain (Figure 2).

## 3. Materials and Methods

### 3.1. Bacterial Strains

The susceptible *A. baumannii* A118 model strain, the isogenic A118 *Δhns* variant, and A118 *Δhns* containing the plasmid pMBLe-*hns*, which expressed a wild-type copy of *hns* under the control of own promoter, were used (Rodgers et al., 2021, submitted). In addition, to extend the role of H-NS in the *A. baumannii* response, the multidrug-resistant and hypervirulent *A. baumannii* AB5075 strain, AB5075 *Δhns* [28], and AB5075 *Δhns* pMBLe-*hns*, were used in the present study.

### 3.2. RNA Extraction and qRT-PCR 

*A. baumannii* A118 and AB5075 and their derivate strains were grown in lysogeny broth (LB) and incubated with agitation for 18 h at 37 °C. Then, a 1:10 dilution in fresh LB broth was realized and incubated for 7 h at 37 °C and 200 rpm. The RNA extraction was performed using the Direct-zol RNA Kit (Zymo Research, Irvine, CA, USA) in triplicate. RNA samples were treated with DNAse (Thermo Fisher Scientific, Waltham, MA, USA) following the manufacturer’s instruction. A PCR amplification of the 16S rDNA gene was performed to verify that DNA contamination was not present. The qRT-PCR was next performed to analyze the expression of natural transformation associated genes. The cDNA was prepared using the iScript™ Reverse Transcription Supermix (BioRad, Hercules, CA, USA) and iQ™SYBR® Green Supermix (BioRad, Hercules, CA, USA) per the manufacturer’s recommendations, respectively. Relative gene expression to *recA* was calculated using the comparative 2^−ΔΔCt^ method [29]. Each cDNA sample was run in triplicate and repeated in at least three independent sets of samples. The ANOVA test followed by Tukey’s comparison was used to determine statistical significance (*p*-value < 0.05) using GraphPad Prism (GraphPad software, San Diego, CA, USA). 

### 3.3. Natural Transformation Assays

Natural transformation assays were performed as described [25,26]. Briefly, 20 uL of *A. baumannii* cells grown overnight in LB medium at 37 °C were mixed with 1 μg of pMBLe-OA-ArK (apramycin resistance) plasmid and the mixture was spotted onto twitching motility plates (10). After 4 h of incubation at 37 °C, the cells were scraped off from the plate and resuspended in a microcentrifuge tube containing 200 μL of LB medium. Apramycin (15 µg/mL) resistant colonies were counted (transformants cells) and scored. In parallel, total colony forming units (CFUs) were performed by plating serial dilutions on LB agar plates. Experiments were conducted in technical and biological triplicates including negative controls. Student’s *t* test analysis was performed using GraphPad Prism (GraphPad software, San Diego, CA, USA). A *p*-value < 0.05 was considered significant.

## 4. Conclusions

The global repressor H-NS modulates the expression of a plethora of *A. baumannii* genes with functions related to virulence, biosynthetic pathways, cell adhesion, quorum sensing, and autotransporters, among others. Previous analyses suggested that H-NS plays a major role in the acquisition of genes transferred horizontally [1,30]. While the acquisition of DNA is a mechanism to better adapt to the environment, as it is the case with numerous mobile genetic elements, too many of these events could have deleterious effects. The acquisition and insertion of DNA fragments into the chromosome were mechanisms that must be tightly regulated to limit the detrimental consequences. H-NS could act as a negative regulator of DNA acquisition.

The focused analysis of the role of H-NS in the regulation of expression of genes related to the mobility and DNA transformation carried out in this analysis showed that: (a) seven genes that code functions associated with natural competence are overexpressed in the absence of H-NS; and (b) the transformation frequency is higher in the absence of this protein. Consequently, H-NS modulates the DNA uptake by *A. baumannii* strains, suggesting a participation in gene acquisition and, concomitantly, evolution during infectious processes.

## Data Availability

All data pertaining to the study described in the manuscript is de-scribed in the report.
